# Baseline cohort data from HARMONY, a cluster randomised controlled trial of culturally safe domestic violence management in general practice

**DOI:** 10.1186/s12875-025-02890-2

**Published:** 2025-07-28

**Authors:** Angela J. Taft, Felicity Young, Kelsey L. Hegarty, Jane Yelland, Danielle Mazza, Douglas Boyle, Richard Norman, Claudia García-Moreno, Cattram Nguyen, Xia Li, Bijaya Pokharel, Molly Allen Leap, Gene Feder

**Affiliations:** 1 Judith Lumley Centre, La Trobe University, Bundoora, Vic 3086 Australia; 2 Department of General Practice and Primary Care, University of Melbourne, Parkville, Australia; 3 Royal Women’s Hospital, Parkville, Australia; 4 Murdoch Children’s Research Institute, Parkville, Melbourne, Australia; 5 Department of General Practice, Monash University, Melbourne, Australia; 6 Curtin University, Perth, WA Australia; 7 Department of Sexual and Reproductive Health and Research, WHO, Geneva, Switzerland; 8 Murdoch Children’s Research Institute, Melbourne, Australia; 9 Department of Paediatrics, University of Melbourne, Parkville, Australia; 10 Statistics Department, La Trobe University, Melbourne, Australia; 11 Centre for Academic Primary Care, Bristol Medical School, University of Bristol, Bristol, England

**Keywords:** General practice, Domestic violence, Cluster randomised controlled trial, Cultural safety, Ethnicity

## Abstract

**Background:**

Domestic violence and abuse (DVA) is a globally prevalent, health damaging problem. In high income countries, migrant /refugee populations from low/middle income countries often consist of young families. DVA is more prevalent, and barriers to disclosure greater among migrant/refugee than native-born families. Consequently, general practice (GP) patient populations are increasingly diverse, but evidence for culturally safe and effective GP management is minimal. The HARMONY study tested a culturally safe DVA intervention to improve GP identification and referral among South Asian families.

**Methods:**

HARMONY was a pragmatic cluster RCT in 19 GP clinics in two regions of Melbourne, Australia. Eligible practices required ≥ 1 South Asian GP; used one of two common software programs; and agreed to have GrHanite™ software on practice computers. This analysis investigated baseline DVA and ethnicity identification in routine electronic GP data. Deidentified aggregated data for female patients aged ≥ 18 with DVA identification, referral, and South Asian ethnicity data were extracted from medical records. Chi Square for comparison of proportions.

**Results:**

Twenty-four clinics were recruited and randomised, but five dropped out due to Covid. Fifty-two percent (50/96) of 19 clinics' staff were South Asian. While 46.7% of female patients (21,220/45,438) were aged 26–45 years, 17.6% (7,874/45,438) were South Asian. There were more South Asian patients in Comparison 20.6% (4,193/20,312) than Intervention 14.7% (3,681/25,126) clinics. South Asian women had less access to Medicare (83% vs 97%) and pensions (13% vs 22%).

At baseline, clinicians recorded 0.58% (265/45,438) DVA-affected women. Notably, they identified fewer South Asian (0.38%) (28/7,874) than non-South Asian women experiencing DVA (0.63%) (237/37,564),—0.28% (0.12%—0.43%), *p* = 0.004. No referrals were identified.

**Conclusion:**

DVA was notably under-identified in these patient populations, but worse among South-Asian female patients. While almost one in six of HARMONY’s female population were South Asian, fewer than four in 1000 South Asian women were identified as experiencing DVA. Greater effort to regularly train and to support GP clinics to better identify DVA is vital but even more important in diverse communities to provide culturally safe DVA identification, care and documentation.

**Trial registration:**

ACTRN12618001845224 on 13/11/2018.

**Supplementary Information:**

The online version contains supplementary material available at 10.1186/s12875-025-02890-2.

## Introduction

Domestic violence and abuse (DVA) is a chronic global epidemic [1]. It can be defined as behaviour that causes psychological, physical, sexual, financial, or emotional harm, and occurs between partners, ex-partners, or other family members. Intimate partner violence, the most common DVA form, is experienced by 23% of Australian women since the age of 15 [2], and affects an estimated 27% of women globally [1]. There is ample evidence that DVA seriously damages women’s mental, physical, psychological, and reproductive health and wellbeing [3]. Women experiencing DVA frequently attend primary care services, however, disclosure rates of DVA to primary healthcare providers has been problematically low, despite victim-survivors having identified these services as a preferred source of support [4, 5].

Over 25% of Australians were born overseas and almost 50% have one or both parents who were born abroad. In origin countries where war or social upheaval recently occurred, few effective sanctions against DVA exist, and where women have no sanctuary, rates of DVA are high [6]. Such countries, including those in South Asia, have significant diaspora communities residing in high-income countries, and are predominantly those with young families, a vulnerable time for DVA. Women from culturally diverse communities, especially migrant and refugee women, experience many barriers to primary care access, which constrains their ability to seek help for DVA in the destination country [7].

In Victoria, Australia, South Asian women are among the largest groups of migrants with young families and are disproportionately represented in DVA agency data [8]. Social isolation, combined with little knowledge of available supports, language barriers, racism and different cultural norms, can leave South Asian women experiencing DVA at great risk of serious harm.

Australia is home to many bilingual/bicultural health professionals, including general practitioners (GPs) who provide care to their own communities. GPs are often the only contact with a professional for a woman who is experiencing DVA [5]. No DVA intervention studies in general practice have specifically addressed the cultural competency of GP clinics. A systematic review of culturally competent DVA responses to women in primary care highlighted the importance of responding to DVA at the system, practice, and provider level [9]. Pokharel et al. recommended an adapted GP systems model, outlining factors for sustainable culturally safe DVA GP practice [9].

This paper reports an analysis of routine GP data from Dec 2018 to Dec 2019, of HARMONY clinics’ characteristics and DVA outcomes at baseline. This period was twelve-months prior to both the intervention and the COVID-19 pandemic. It investigates routine recorded DVA care in all female patients, but especially those from South Asian communities.

## Methods

HARMONY was conducted in GP clinics in North-West and South-Eastern Melbourne with high proportions of South Asian communities.

### Design and objectives

HARMONY was designed as a pragmatic (parallel group) cluster-randomised controlled trial to test the effectiveness of a DVA GP clinic system intervention, described in the protocol paper [10], with a focus on cultural competency and South Asian patients. HARMONY aimed to increase DVA identification and referral (primary outcomes) among women attending Intervention vs Comparison GP clinics [10].

### Eligibility criteria

GP clinics needed to: (a) have at least one South Asian bilingual/bicultural GP; (b) use either one of two most common GP medical software programs; and (c) agree to have anonymised data extraction from routine electronic medical records by the GrHanite™ software program. All 24 recruited clinics gave informed consent through a signed Memorandum of Understanding.

### Randomisation

Upon rolling recruitment of ≥ 10 from a sample of 24 clinics and GrHanite™ installation [11], a statistician (blind to group) used WINPEPI (PEPI-for-Windows) computer minimisation program, to randomly allocate clinics to Intervention or Comparison group.

Clinics were randomised, stratified by:

- ❖ size—small (≤ 5 doctors) and large (≥ 6 doctors),
- ❖ Socio-Economic Indexes for Areas (SEIFA) for clinic postcode [12, 13] (1- 5 classified as'lower, more disadvantaged’ and 6 or more as'higher, more advantaged) and
- ❖ North-West or South-East region.

### DVA measure

An algorithm to identify women with indications of DVA was developed by DB, KH, AT and FY. This algorithm is the subject of a separate paper under development; however, the following mechanisms were used.

- A cohort of anonymised patient free text notes from the HARMONY pilot female patient population were manually reviewed by an expert DVA GP researcher (KH). She classified data from these notes into the following categories:
  i. Probable relationship issues
  ii. Possible mental health presentation
  iii. Possible non-mental health presentation
  iv. Abuse by a stranger
  v. Definite DVA
  vi. Past DVA
  vii. Visit to a psychologist
- In the pilot cohort selected, 15% of the records were classified under categories, iv, v and vi.
- Term Frequency Analysis was utilised to determine words associated with category (v) (Definite DVA). Using these DVA-associated free text terms, human review allowed us to classify the importance of specific terms and combinations allow weighting of risk to be determined. Validating different numbers of key words and weightings allowed the conversion of the term frequency analysis into an SQL-based predictive algorithm designed to classify records as potentially meeting classification (v). Within the validated cohort, co-location was utilised to trace words commonly appearing next to each other or in a related location. The algorithm was refined with Text classification to ensure it classified records correctly from the validated dataset for category (v) and had minimal false-positives from other categories. Manual review identified challenges in separating Abuse by a stranger (iv) and past DVA (vi) from definite DVA (v).
- This initial algorithm was tested against a larger patient record cohort that had not been manually validated. DVA according to the algorithm was reviewed, and a false positive rate of 14% was identified, however, in almost all cases there was an associated risk of DVA, although some records indicated DVA in other family members or associated mental health issues. Because the same algorithm was applied to the intervention and comparison populations, a false positive DVA will dilute the difference in DVA characteristics between intervention and control. This introduces noise leading to a weaker signal between intervention and comparison.

### South Asian measure

South Asia comprises a diverse range of populations and naming practices. We collected surnames/family names used in India, Nepal, Afghanistan, Sri-Lanka, Pakistan, and Bangladesh with a comprehensive process outlined in our protocol [10]. Research staff finalized a list of 7690 verified surnames from the South Asian countries above. Limitations are discussed below. These surnames were flagged in the GrHanite™ software, so we could estimate the overall South Asian female patient population and the proportion in each clinic and study arm. These data were combined with the little ethnicity data recorded by GP clinic staff.

### Data extraction

GrHanite™ was designed by the University of Melbourne to help manage GP electronic data acquisition for audit, research and health surveillance [11]. Study data related to eligible ‘active’ female patients aged ≥ 18 years defined by the Royal Australian College of General Practice as *three or more visits in the last two years *[14].

Prior to the HARMONY intervention, IT staff installed GrHanite™ within each clinic. Data from electronic medical records were extracted using GrHanite™, not only de-identified before extraction – but aggregated – no individual level data were extracted. At installation, GrHanite™ extracted historical data (December 2018 to December 2019) from all recruited GP clinics, which form this paper’s data.

#### Consent and confidentiality

As this study was a cluster trial, we obtained consent at the GP practice level. Given the nature of the data collected, i.e., patient identifiers removed (deidentified), and the privacy protecting techniques used by GrHanite™ to protect individual identity, consent to query the patient electronic medical records was sought only from the general practices, and not individual patients. Given the protections in place, the project met ethics and requirements and rationale to be granted a waiver of consent.

### Data analysis

The proportions and corresponding 95% confidence intervals (CIs) were calculated using the function ‘binconf’ from the package ‘Hmisc’ in R with Wilson confidence intervals [15]. To compare two proportions between groups, we used R function ‘prop.test’ for Chi-square.

### Ethics

Approval granted by La Trobe University Human Ethics Committee (HEC18413) and research conducted according to the National Health and Medical Research Council National Statement on Ethical Conduct in Human Research.

## Results

HARMONY recruited and randomised 24 clinics, however five dropped out due to shortage of staff and high COVID-19 rates (see Supplementary Fig. 1—Consort flow chart). This paper includes data from all 19 retained clinics. It covers the baseline period (1 December 2018 −1 December 2019) for 18 clinics. One clinic’s data are from 1 December 2019—1 December 2020, because it was established in 2019.

### Characteristics of GP clinics

There were more small clinics (≤ 5 GPs) than large overall and one more in the Intervention than Comparison group. The region’s clinics were roughly equal. Over half of the clinic staff were South Asian (52%), but Intervention clinics had a lower proportion of South Asian staff and a higher proportion of GP clinics in disadvantaged (60%) areas than Comparison clinics (44%) (Table 1).

**Table 1:** General practice clinic characteristics

	Comparison	Intervention	Total
	(*n* = 9)	(*n* = 10)	(*n* = 19)
Size			
Small ≤ 5 General Practices	6	9	15
Large ≥ 6 General Practices	3	1	4
Region			
North-West Melbourne	5	5	10
South-East Melbourne	4	5	9
SEIFA (index of dis/advantage)			
Lower (1–5)	4	6	10
Higher (6–10)	5	4	9
Total Clinicians *(% South Asian)*	49 *(63.3%)*	47 *(40.4%)*	96 *(52.1%)*
	*30 South Asian*	*20 South Asian,*	*50 South Asian,*
	*19 non-South Asian*	*27 non South Asian*	*46 non South Asian*

### Female patient characteristics

Table 2 outlines the characteristics of 45,438 active female patients in the 19 clinics (mean 2,398 per clinic). Women of reproductive age represented the highest proportion (26- to 45). There was a higher proportion of South Asian patients in Comparison (4193/20312, 20.6%) than Intervention group (3681/25216, 14.7%).

**Table 2:** Female patient demographics

	Comparison	Intervention	Total
	*N* = 20312 (%)	*N* = 25126 (%)	*N* = 45438
Age			
18–25	2374 (11.7)	3553 (14.1)	5927
26–35	5498 (27.1)	6589 (26.2)	12087
36–45	4483 (22.1)	4650 (18.5)	9133
46–55	2960 (14.6)	3643 (14.5)	6603
56–65	2273 (11.2)	2957 (11.8)	5230
≤ 66	2724 (13.4)	3734 (14.9)	6458
Medicare			
No	1253 (6.2)	1239 (4.9)	2492
Yes	19059 (93.8)	23887 (95.1)	42946
Pension			
No	16396 (80.7)	19482 (77.5)	35878
Yes	3916 (19.3)	5644 (22.5)	9560
Private Health Insurance			
No	19701 (97.0)	24830 (98.8)	44531
Yes	611 (3.0)	296 (1.18)	907
Health Care Concession			
No	16861 (83.0)	20542 (81.7)	37403
Yes	3451 (17.0)	4584 (18.2)	8035
South Asian patients			
No	16119 (79.4)	21445 (85.4)	37564
**Yes**	**4193 (20.6)**	**3681 (14.7)**	**7874**
SEIFA			
1–3	2491 (12.3)	3788 (15.1)	6279
4–7	8948 (44.1)	14559 (57.9)	23507
**8–10**	**8680 (42.8)**	**6496 (25.9)**	**15176**
No Postcode recorded	193 (1.0)	283 (1.1)	476

While most patients reported access to Medicare (Australia’s health insurance scheme), around 5% (6.2% Comparison, 4.9% Intervention) did not. Around one in six women (17.6%) had Health Care Concession Cards and slightly more Intervention group women lived in SEIFA disadvantaged areas (15.1%) than Comparison (12.3%).

### Female patients recorded as experiencing DVA

The overall Comparison DVA identification rate was 100/20312, 0.0.49% (95% confidence intervals (CI) 0.4%—0.6%) and the Intervention group was 165/25126, 0.66% (95% CI 0.6%—0.8%). The difference between rates was −0.2% (−0.3% to −0.03%).

Like the overall patient population, the majority of DVA victim-survivors in both groups were in the reproductive age group (26–45 years). The percentage of recorded DVA victim-survivors without access to Medicare at baseline was smaller (1.6%) than the overall female patient population (5.6%). However, more DVA recorded women were on a pension, and had access to Health Care Concession cards (Table 3).

**Table 3:** Demographics of all female patients recorded as having experienced/experiencing DVA

Demographic information	Comparison	Intervention	Total	*p* Value
	*N* = 100/20,312	*N* = 165/25,126	*N* = 265/45,438	
	0.49%	0.66%	0.58%	
Age				0.94
18–25	13 (13%)	23 (14%)	36	
26–35	31 (31%)	47 (29%)	78	
36–45	25 (25%)	39 (24%)	64	
46–55	20 (20%)	32 (19%)	52	
56–65	6 (6%)	10 (6%)	16	
66 ≤	5 (5%)	14 (9%)	19	
Medicare				1.0
Yes	98 (98%)	163 (99%)	261	
No	2 (2%)	2 (1%)	4	
Pension				0.16
Yes	40 (40%)	52 (32%)	92	
No	60 (60%)	113 (69%)	173	
Private Health				0.99
Yes	1 (1%)	3 (2%)	4	
No	99 (99%)	162 (98%)	261	
Health Care Concession				0.29
Yes	36 (*36%*)	49 (*30%*)	85	
No	64 (64%)	116 (70%)	180	
SEIFA				0.13
1–3	12 (12%)	15 (9%)	27	
4–7	52 (52%)	107 (65%)	159	
8–10	34 (34%)	41 (25%)	75	
No postcode	2 (2%)	2 (1%)	4	
South Asian	16 (16%)	12 (7%)	28	

NB. Because of aggregated data we were unable to compare DVA to non-DVA patients, only to the overall population

#### DVA GP recorded identification rate of South Asian women

While 265 women were recorded as experiencing DVA overall (0.58%), a significantly lower proportion of South Asian (0.38%) versus non South Asian DVA (0.63%) patients was recorded overall and by each arm.

Table 4 below demonstrates how proportionally fewer South Asian victim/survivors were recorded than those who were not South Asian.

**Table 4:** DVA identification in general practice records by South Asian ethnicity

				Total
Baseline female patients in all clinics (*n* = 45,438)	South Asian DVA identified (*n* = 28/7874)	Non-South Asian DVA identified (*n* = 237/37564)	*Difference (95% CI and p-value)*	All DVA identified (*n* = 265/45,438)
Comparison clinics (all female patients, *n* = 20,312)	16/4173 (0.38%)	84/16,139 (0.52%)	0.14% (−0.08%—0.35%) *p* = 0.26	100/20,312 (0.49%)
Intervention clinics (all female patients, *n* = 25,126)	12/3681 (0.33%)	153/21,445 (0.71%)	0.39% (0.17%—0.60%) *p* = 0.007	165/25,126 (0.66%)
Total	28/7874 (0.38%)	237/37,564 (0.63%)	0.28% (0.12%—0.43%) *p* = 0.004	265/45,438 (0. 58%)
Difference (95% CI) between identification of DVA among South Asian versus Non-South Asian female patients	−0.06% (−0.32%- 0.21%) *P* = 0.67	0.19% (0.03%—0.35%) *p* = 0.02		

Comparison clinics identified 0.38% South Asian DVA-affected, but 0.52% non-South Asian women and this was not significantly different. However, Intervention clinics identified 0.71% non-South Asian, but significantly fewer DVA-affected South Asian women (0.33%).

There were *no referrals* for any woman recorded for either group.

## Discussion

### Summary

HARMONY is the first Australian trial to use routine GP electronic data to estimate the proportion of DVA affected women reported among a large and diverse sample of female GP patients. We highlight the very low baseline of DVA identification documented by clinics who chose to remain in a cultural competency DVA trial during the COVID-19 pandemic. More notably, we report the first estimated comparison of those recorded with DVA among non-South Asian and South Asian female patients, one of the largest diaspora migrant groups in Australia and the UK. We demonstrate that identification of South Asian victim/survivors is notably small, despite the increased risks in this population.

### Strengths and limitations

There are limitations to our DVA algorithm development, as it was based on a limited number of case notes and requires further testing. For South Asian estimates, there are many limitations. Cultural identity and experience may differ from ethnic origin. An individual with an ethnic name who has multi-generational roots within Australia may be very different, compared to one with a non-ethnic name, whose parents emigrated to Australia. We will not have picked up South Asian women who married non-South Asian men and changed their names. There could be other naming practices that we overlooked. However, we took the decision to be more inclusive rather than less, as we were unaware of any other possible method to estimate this population. Nevertheless, as the proportion of South Asian women was roughly approximate to those in the overall population, we suggest this indicates a probable DVA under-reporting of DVA in general and DVA among South Asian female patients in the overall Australian GP clinic population.

While the methodology for the DVA algorithm and South Asian estimates has limitations, this large baseline sample analysis suggests the possible under-identification of DVA, especially among migrant and refugee groups in general practice. In the most recent Australian Bureau of Statistics Personal Safety Survey (2021–22) [16], 27% of women aged 18 + reported DVA since the age of 15, while the reported 12-month prevalence rate dropped from 2.3% to 1.5%. In these HARMONY data, many fewer (overall 0. 58%) victim/survivors were identified in a GP clinic setting, where one would expect a higher proportion of women experiencing DVA to attend, due to their worse health, compared to non-abused women [3, 17].

### Comparison with existing literature

The English trial on which HARMONY is based (IRIS) [18] also found a low rate of 0.3% women identified in both arms at baseline. The only two previous Australian DVA GP prevalence studies conducted, surveyed women directly in GP clinics. Hegarty et al. (2002) found that 10% of female respondents reported experiencing DVA in the last twelve months and 37% over a lifetime [17]. Mazza et al. (1996) found that over a quarter of women in relationships had been victims of physical or emotional partner abuse in the previous year, with one in 10 having experienced severe physical violence [19]. However, HARMONY data indicate that the recorded DVA identification rates are markedly below the rates reported in these earlier studies. Due to different methods used in a waiting room survey, there is highly likely to be a high rate, compared with GP electronic records [20].

There may be many reasons for this very low rate of DVA identification. First, documentation of DVA may be prone to underreporting due to our algorithm. Further reasons may rest with both clinicians and women. A recent meta synthesis found that GPs may feel unprepared for the task of DVA care and therefore are reluctant to ask or document it in records [21]. DVA clinician training generally raises clinicians' level of readiness and confidence, but evidence of their behaviour change is limited [22]. A further meta synthesis found that women may not disclose if not asked sensitively and specifically [5]. Finally, it may be a true rate.

HARMONY was conducted in areas chosen for their high South Asian populations and found that these GPs saw approximately one in five female South Asian patients. However, while GPs identified fewer than one in a hundred female patients as DVA victim/survivors, they identified fewer among South Asian women, while South Asian women were more than a sixth of their female patient population. This is a significant finding as South Asian women are likely to be more at risk of severe harm than non-South Asian women, due to the many significant barriers to their disclosing and attaining the help that exists in the wider society, in their own communities and in their families [23]. We were dismayed to find no referrals documented, but GPs may not know where to refer women. This is a common difficulty reported by GPs [21]. GP clinics may be one of the few sanctioned places where families permit South Asian women to go, so that an effective and supported culturally safe GP clinic response could be critical to ensure women’s safety and well-being [24].

### Implications for research and practice

These baseline data demonstrate the necessity of a much more sustained effort for health system reform to support GPs to identify and document women experiencing DVA among diverse patient populations. One way to do this is to provide regular effective training and resources to GP clinics to accurately identify and safely document and support all victim/survivors in their patient populations. Culturally safe methods are an essential training component to ensure that the identification of migrants and refugee victims/survivors is significantly improved.

## Supplementary Information

Supplementary Material 1.

## Data Availability

Data that support the findings of this study have been deposited in the OPAL Figshare database at La Trobe University. Data collected for the HARMONY trial, including deidentified aggregated participant data and a data dictionary defining each field in the set, are available to scholars on request from OPAL storage. • La Trobe research repository-including Harmony data and analysis guides (deidentified participant data) https://opal.latrobe.edu.au/articles/dataset/Harmony_data_and_analysis_guides/26892316. • Data will be made available with investigator support, after approval of a proposal, with a signed data access agreement.
