# Supplementary material for: Baseline cohort data from HARMONY, a cluster randomised controlled trial of culturally safe domestic violence management in general practice
Source: BMC Prim Care. 2025 Jul 28;26:232. doi: 10.1186/s12875-025-02890-2 (PMC12302802; doi:10.1186/s12875-025-02890-2)
Supplement: Supplementary file 1 — Supplementary Material 1. [file 12875_2025_2890_MOESM1_ESM.docx]

Supplementary Figure 1 Harmony Consort Flowchart

No clinics withdrew

No clinics withdrew
